# *NMRpQuant*: an automated software for large scale urinary total protein quantification by one-dimensional ^1^H NMR profiles

**DOI:** 10.1093/bioinformatics/btac502

**Published:** 2022-07-21

**Authors:** Panteleimon G Takis, Ivan Vuckovic, Tricia Tan, Aleksandar Denic, John C Lieske, Matthew R Lewis, Slobodan Macura

**Affiliations:** Section of Bioanalytical Chemistry, Division of Systems Medicine, Department of Metabolism, Digestion and Reproduction, Imperial College London, London SW7 2AZ, UK; National Phenome Centre, Department of Metabolism, Digestion and Reproduction, Imperial College London, London W12 0NN, UK; Metabolomics Core, Mayo Clinic, Rochester, MN 55905, USA; Division of Diabetes, Endocrinology and Metabolism, Department of Metabolism, Digestion and Reproduction, Imperial College London, London W12 0NN, UK; Clinical Biochemistry, Blood Sciences, North West London Pathology, Charing Cross Hospital, London W6 8RF, UK; Division of Nephrology and Hypertension, Department of Internal Medicine, Mayo Clinic, Rochester, MN 55905, USA; Division of Nephrology and Hypertension, Department of Internal Medicine, Mayo Clinic, Rochester, MN 55905, USA; Section of Bioanalytical Chemistry, Division of Systems Medicine, Department of Metabolism, Digestion and Reproduction, Imperial College London, London SW7 2AZ, UK; National Phenome Centre, Department of Metabolism, Digestion and Reproduction, Imperial College London, London W12 0NN, UK; Metabolomics Core, Mayo Clinic, Rochester, MN 55905, USA; Department of Biochemistry and Molecular Biology, Mayo Clinic, Rochester, MN 55905, USA

## Abstract

**Summary:**

^1^H nuclear magnetic resonance (NMR) spectroscopy is an established bioanalytical technology for metabolic profiling of biofluids in both clinical and large-scale population screening applications. Recently, urinary protein quantification has been demonstrated using the same 1D ^1^H NMR experimental data captured for metabolic profiling. Here, we introduce *NMRpQuant*, a freely available platform that builds on these findings with both novel and further optimized computational NMR approaches for rigorous, automated protein urine quantification. The results are validated by interlaboratory comparisons, demonstrating agreement with clinical/biochemical methodologies, pointing at a ready-to-use tool for routine protein urinalyses.

**Availability and implementation:**

*NMRpQuant* was developed on MATLAB programming environment. Source code and Windows/macOS compiled applications are available at https://github.com/pantakis/NMRpQuant, and working examples are available at https://doi.org/10.6084/m9.figshare.18737189.v1.

**Supplementary information:**

Supplementary data are available at *Bioinformatics* online.

## 1 Introduction

Human urine is one of the most studied biofluids in clinical and population scale biomedical research. Despite its complexity—containing >2000 metabolites (Bouatra *et al.*, 2013; Takis *et al.*, 2017)—urinalysis by nuclear magnetic resonance (NMR) requires only minimal sample handling and produces rich compositional data which reflect the metabolic phenotype of the sample donor. Protein is present in minute quantities in healthy urine but is often a key indicator of kidney disease when elevated (i.e. proteinuria) (Garg *et al.*, 2002), and therefore, its quantification is of clinical importance for detecting and monitoring disease.

**Fig. 1.**
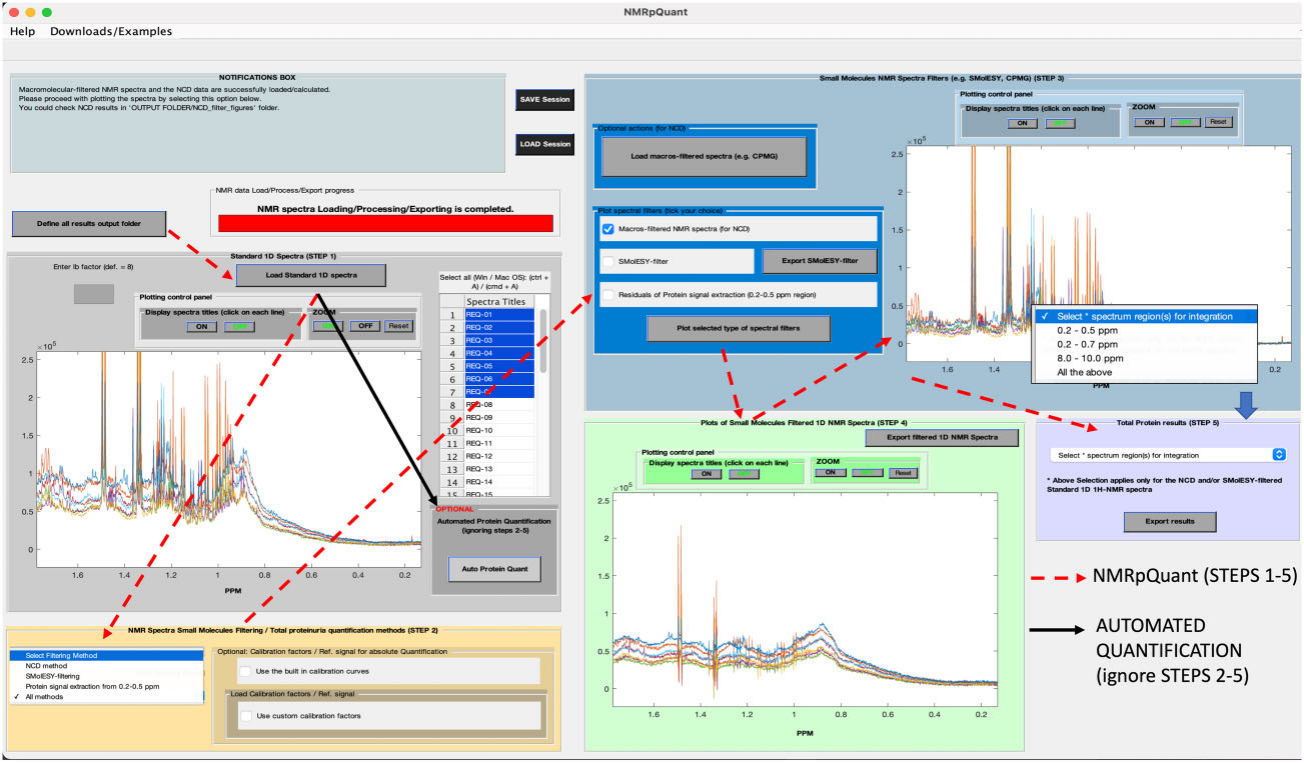
NMRpQuant interface. GUI overview/functionalities/plots (dashed arrows) for automated protein quantification via proteinuric ^1^H NMR profiles, consisting of 5 ‘button-click’ steps (from left to right): loading of NMR spectra (Step 1), selection of metabolites’ signals filtering method and the type of total protein quantification (Step 2), visualization of the filtering method data (right plotting panel) (Step 3) as well as of the filtered spectra (bottom plotting panel) (Step 4) and selection of filtered spectral region(s) for integration as well as exportation of relative or absolute total protein concentration values (Step 5). Black arrow highlights the option of automated quantification by SMolESY-based filtering, skipping Steps 2–5

^1^H NMR is widely used for biomarker detection in metabolic profiling studies of biofluids, providing highly reproducible quantitative measurements of small metabolites (Keun *et al.*, 2002). Protein is also detectable by ^1^H NMR via its broad peaks owing to the plethora of protons and their overlapped NMR signals, resonating across whole spectral range (Takis *et al.*, 2020). The quantification of urinary protein by integration of selected protein signal regions—after filtering out metabolite ^1^H NMR signals via additional NMR experiments or Small Molecule Enhancement SpectroscopY (SMolESY) (Takis *et al.*, 2020)—was recently reported (Vuckovic *et al.*, 2021), allowing simultaneous small molecule and protein quantification. Building on this manual approach, which requires strong mathematical/NMR expertise, we developed *NMRpQuant* (Fig. 1), an automated and freely available software for urinary protein quantification from standard ^1^H NMR profiles. Automated spectral region integration and metabolite signal suppression (i.e. filtering)—including a new approach—are implemented to deliver readily interpretable total urine protein concentration measurements without the need for manual interrogation of the NMR spectra. *NMRpQuant* is therefore directly implementable in both clinical and high throughput NMR-based urine metabolomics applications (e.g. population screening and molecular epidemiology). Software performance was validated in multi-centered urine cohorts versus clinical results from independent laboratories, ensuring reliable and accurate results.

## 2 NMRpQuant

### 2.1 Implementation

*NMRpQuant* is an open-source MATLAB GUI software. It is also compiled for Windows (.exe) and macOS (.app) operating systems (without MATLAB dependencies), having been employed on Windows 10/macOS Sierra (10.12) and above (https://github.com/pantakis/NMRpQuant). Emphasis was given to a user-friendly and interactive interface for visualizing both raw NMR spectra and filtered data, performance of metabolite signal filtering, and facilitating parameter optimization. Built in parameters for protein quantification require the presence of Bruker IVDr reference signal (ERETIC), however, independent parameters/reference signal(s) can be used instead (Supplementary Material).

### 2.2 Features—results—validation

#### 2.2.1 Inputs—metabolites signals filtering

*NMRpQuant* requires Bruker 1D ^1^H NMR spectral data [acquired/Fourier-transformed/phase and baseline corrected under common urine metabolomics standard operating procedures (SOPs) (Supplementary Material)]. The software reads the absorption (1r), dispersion (1i) and FID (fid) spectral data. It automatically suppresses (filters out) metabolite NMR signals: (i) by protein methyl signals extraction from 0.2 to 0.5 ppm, (ii) SMolESY method and (iii) 1D NOESY (i.e. standard ^1^H NMR experiment) and CPMG spectra Difference (NCD) (Fig. 1) (Supplementary Material). The automated SMolESY-filtering is a key feature, reducing acquisition time and cost, since NCD requires acquiring an additional CPMG spectrum.

#### 2.2.2 Total protein quantification

Users can select a metabolite signal depleted spectral region for protein quantification and integrate among 0.2–0.5, 0.2–0.7 (parts of protein methyl groups) and 8.0–10.0 ppm (part of aromatic/amide protein groups) (or all) options. The latter could allow as good as or better quantification of total protein (in mg/ml) compared to individual region integration (Supplementary Material) (Vuckovic *et al.*, 2021).

#### 2.2.3 Validation/evaluation of performance

**Fig. 2.**
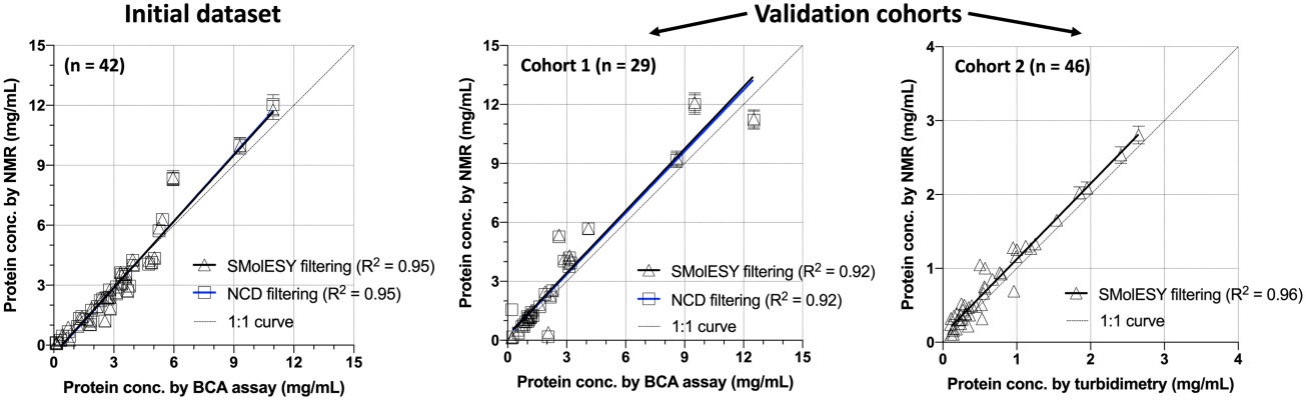
NMRpQuant performance. The software was built on a 42 urine samples cohort (Vuckovic *et al.*, 2021) (left panel) and it was tested on two multicenter validation cohorts consisting of 29 (middle panel) and 46 urine samples (right panel). Independent protein measurements were also carried out via different biochemical methods (i.e. BCA and turbidimetry) (see Supplementary Material). Results (*R*^2^ > 0.9) showed a very good agreement between *NMRpQuant* and clinically/biochemically measured concentrations of total protein in urine

The parameters for absolute quantification and metabolite signal filter optimization were built upon a set of 42 urine sample 1D ^1^H NMR spectra and Bicinchoninic acid (BCA) protein analyses (Fig. 2). Raw NMR and protein data could be downloaded from: https://doi.org/10.6084/m9.figshare.18737189.v1 and corresponding software outputs from https://doi.org/10.6084/m9.figshare.18737372.v1. Further validation was performed in cohorts containing 46 and 29 urine samples with total protein assessed by turbidimetric and BCA methods, respectively. Urine NMR spectra were recorded at independent laboratories using the same SOPs (Supplementary Material). *NMRpQuant* results linearly correlated with the traditional total protein measurements (*R*^2^ > 0.9) (Fig. 2), suggesting that NMR is an acceptable alternative (Supplementary Material).

#### 2.2.4 Applicability—current limitations

*NMRpQuant* requires only the standard 1D ^1^H NMR (NOESY-1D) metabolomics spectra (and optionally CPMG spectra if the NCD method is used) (see SOPs in Supplementary Material). This allows retrospective quantification of protein in urine from NMR metabolomics legacy datasets. Notably, *NMRpQuant* allows the use of any alternative reference compound (ERETIC is the default one) for total protein absolute quantification. The software was built on the cohort of 42 patients (with focal segmental glomerulosclerosis and total protein concentrations known from independent BCA analysis) and validated by two independent adult cohorts consisting of patients suffering from various pathologies (e.g. viral infections, chronic kidney disease etc.) (see Fig. 2 and Supplementary Material). In principle, *NMRpQuant* is generalizable to any liquid mixture of proteins and small molecules (e.g. blood plasma, protein-based pharmaceutical formulation, etc.). However, each mixture needs independent calibration as the shape and intensity of the protein part of the spectrum depends on the environment. A limitation of the method is that its sensitivity is limited to higher than ∼0.1 mg/ml of total protein concentration. Additionally, at lower protein concentrations (less than ∼0.5 mg/ml), its accuracy is affected by small imperfections in metabolites’ signals suppression.

## 3 Conclusions

*NMRpQuant* is an automated platform for the total quantification of protein in urine using standard 1D ^1^H NMR spectra, validated in independent cohorts against independent traditional total protein measurements. It requires minimal NMR or computational skills and is ideal for retrospective application to NMR-based metabolomics data for total urinary protein quantification without additional testing.

## Supplementary Material

btac502_Supplementary_Data
