# Supplementary material for: NMRpQuant: an automated software for large scale urinary total protein quantification by one-dimensional 1H NMR profiles
Source: Bioinformatics. 2022 Jul 21;38(18):4437–9. doi: 10.1093/bioinformatics/btac502 (PMC9477529; doi:10.1093/bioinformatics/btac502)
Supplement: btac502_Supplementary_Data [file btac502_supplementary_data.pdf]

SUPPLEMENTARY MATERIAL

NMRpQuant, an automated software for large scale urinary total protein quantification by one-dimensional ^{1}H NMR profiles

Panteleimon G. Takis[1,2,*], Ivan Vuckovic[3], Tricia Tan[4,5], Aleksandar Denic[6], John C. Lieske[6], Matthew R. Lewis[1,2] and Slobodan Macura[3,7]

[1]Section of Bioanalytical Chemistry, Division of Systems Medicine, Department of Metabolism, Digestion and Reproduction, Imperial College London, South Kensington Campus, London SW7 2AZ, UK
[2]National Phenome Centre, Department of Metabolism, Digestion and Reproduction, Imperial College London, Hammersmith Campus, IRDB Building, London W12 0NN, UK
[3]Metabolomics Core, Mayo Clinic, Rochester, MN 55905, USA
[4]Division of Diabetes, Endocrinology and Metabolism, Department of Metabolism, Digestion and Reproduction, Imperial College London, London W12 0NN, UK
[5]Clinical Biochemistry, Blood Sciences, North West London Pathology, Charing Cross Hospital, London W6 8RF, UK
[6]Division of Nephrology and Hypertension, Mayo Clinic, Rochester, MN 55905, USA
[7]Department of Biochemistry and Molecular Biology, Mayo Clinic, Rochester, MN 55905, USA

*To whom correspondence should be addressed. E-mail: p.takis@imperial.ac.uk

Contents

1. NMR urine samples preparation/acquisition details (SOPs) *S3*

2. Biochemical/clinical proteinuria measurements details *S4*

2.1 BCA assay **S4**

2.2 Turbidimetric method **S4**

3. Proteinuria: How it could be detected by standard 1D ^{1}H NMR urine profiles? *S5*

4. NMRpQuant platform *S7*

4.1 The general purpose **S7**

4.2 Metabolites ^{1}H NMR signals removal (filtering) methods **S7**

4.2.1 NCD method (optional) S7

4.2.2 SMolESY filtering S8

4.2.3 Protein (broad) signals extraction from 0.2–0.5 ppm spectral region S9

4.3 Automated spectral regions integration – Total protein absolute quantification **S10**

5. Guidelines plus detailed Graphical User Interface (GUI) description *S12*

5.1 NOTIFICATIONS BOX – Output folder – Spectra loading/processing progress bar **S12**

5.2 STEP 1 .... **S13**

5.3 STEP 2 .... **S14**

5.4 STEP 3 .... **S15**

5.5 STEP 4 .... **S17**

5.6 STEP 5 .... **S17**

5.7 Prerequisites/Important notes for successful implementation of *NMRpQuant* **S19**

5.7.1 NMR spectra preparation/input files S19

5.7.2 Urine samples preparation S19

5.7.3 Notes for SMolESY-based filtering – Protein extraction signals from 0.2–0.5 ppm region S20

5.7.4 Notes for total urinary protein absolute quantification S20

5.8 Save/Load an *NMRpQuant* session (save/load checkpoint) **S20**

6. NMRpQuant: performance in automated mode *S21*

6.1 Initial urine samples cohort: NMR vs BCA total protein quantification results **S21**

6.2 Multicentered validation urine samples cohorts: NMR vs (BCA & clinical methods) total protein quantification results **S23**

7. Availability of Raw NMR data and biochemical protein measurements – Input/Output files *S26*

8. Technical Requirements *S27*

8.1 MATLAB dependencies – Operating Systems **S27**

8.2 Recommended computational resources **S27**

9. Supplementary References *S28*

1. NMR urine samples preparation/acquisition details (SOPs)

The general procedure of NMR samples preparation is described in detail in Dona et al.(Dona *et al.*, 2014) Briefly, NMR samples were prepared into 96-well plates by adding 630 µL of urine sample to each well of the 96-well plate and mixed with 70 µL of urine buffer [urine buffer: 1.5 M KH_2PO_4 dissolved in 99.9% ^2H_2O, pH 7.4, 2 mM NaN_3 and 5.8 mM 3-(trimethyl-silyl)propionic acid-d_4 (TSP)]. 600 µL of the mixture was transferred into NMR tubes. Solution ^1H NMR spectra of all samples were acquired using a Bruker IVDr 600 MHz spectrometer (Bruker BioSpin) operating at 14.1 T and equipped with a 5 mm PATXI H/C/N (Imperial) or 5 mm BBI (Mayo) S3probe (MAYO) with ^2H-decoupling including a z-axis gradient coil, an automatic tuning-matching (ATM) and an automatic refrigerated sample changer (SampleJet). Temperature was regulated to 300 ± 0.1 K. For each urine NMR sample, two NMR experiments were acquired in automation: a general profile ^1H NMR water presaturation experiment using a one-dimensional pulse sequence where the mixing time of the 1D-NOESY experiment is used to introduce a second presaturation time and a 2D *J*-resolved experiment. The FIDs were multiplied by an exponential function equivalent to 0.3Hz line-broadening before applying Fourier transform. All Fourier transformed spectra were automatically corrected for phase and baseline distortions and referenced to the TSP singlet at 0 ppm. For quality control assessment a pooled QC sample was similarly prepared by combining equal parts of each study sample and pooled QC samples were acquired regularly throughout the sample analysis.

CPMG spectra were recorded using a standard pulse sequence (cpmgpr1d; Bruker BioSpin) with acquisition and processing parameters matching those of 1D NOESY spectra. The spin-echo time was 600 µs with a train of 128 refocusing pulses.

2. Biochemical/clinical proteinuria measurements details

2.1 BCA assay

A 100 μL aliquot of freshly thawed urine was buffer-exchanged using 0.5 mL Zeba desalting column (Thermo Fisher Scientific) previously equilibrated in 20 mM ammonium bicarbonate buffer, a volatile buffer. This step is necessary to remove contaminating substances in urine and allow the concentration of low-protein samples. All sample processing was performed at 4 °C. The desalted samples were frozen and dried down using a centrifugal vacuum concentrator (SpeedVac, Savant). Samples were resolubilized with 50 μL of 0.1% sodium dodecyl sulfate (SDS)/20 mM Tris buffer, pH 8.5, and heated at 85 °C for 10 min to fully solubilize and denature proteins. Protein concentrations were determined in replicate protein assays (BCA, Thermo Fisher Scientific) with at least two dilutions per sample in duplicate, using the microplate method according to the manufacturer's instructions. All samples and the denatured bovine serum albumin standard were diluted in SDS buffer for the assays. This assay is compatible with SDS up to 5% v/v. Data were acquired, and protein concentrations were calculated with a SpectraMax Plus microplate reader and SoftMax Pro software (Molecular Devices).

2.2 Turbidimetric method

Initially, protein denaturation in 46 urine samples was accomplished with benzethonium chloride, using 07P59 Alinity c Urine/CSF Protein Reagent Kit, Abbott. Total protein in each urine sample was quantified turbidimetrically with the Abbott Alinity ci analyser, using the wavelength of 404 nm at North West London Pathology, UK (Yılmaz *et al.*, 2004).

3. Proteinuria: How it could be detected by standard 1D ^{1}H NMR urine profiles?

Urine is known to be a very complex mixture of small molecules (i.e. metabolites) along with proteins (Bouatra *et al.*, 2013). Urine is mostly dominated by metabolites (Takis *et al.*, 2017), with healthy urine protein excretion <200 mg/day (Lamb *et al.*, 2009). However, pathological conditions could cause kidney's damage, lowering its protein filtration capacity/ability causing increase of protein concentration (proteinuria). Clinically, the proteinuria detection as well as accurate quantitation is a significant diagnostic tool for renal malfunction (Lamb *et al.*, 2009).

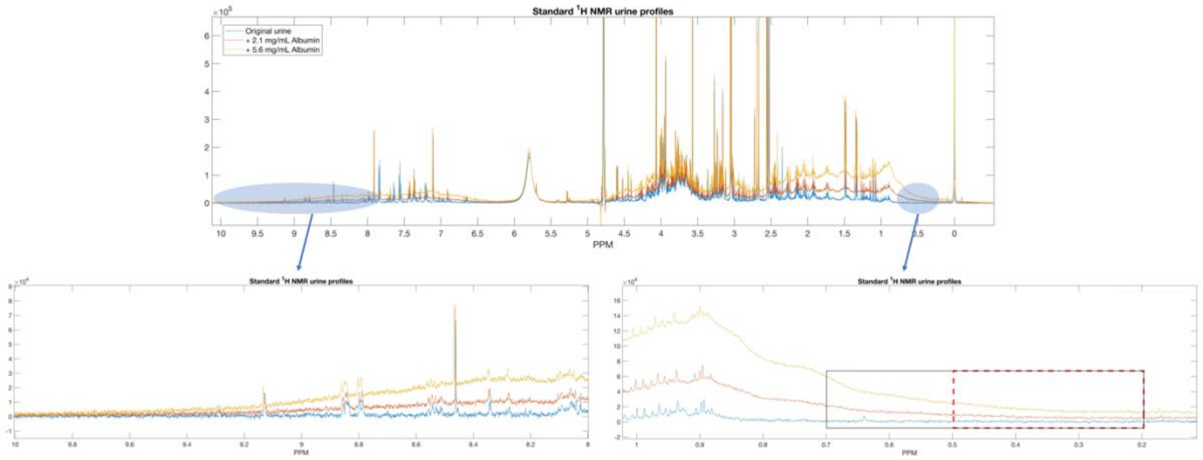

Figure S1. Top panel shows three urine 1D ^{1}H NMR profiles from the same urine sample (blue line) spiked with two different concentrations of albumin (yellow – orange lines). Bottom panels focusing on the three spectra regions of the urine NMR spectra that capture parts of methyl and aromatic/amide protons from proteins in urine and are automatically integrated by *NMRpQuant*.

The most common methods for the total protein quantification in urine are colorimetric [i.e. bicinchoninic acid (BCA) assay (Lamb *et al.*, 2009) etc.] due to their low cost, high sensitivity and high-throughput (Yılmaz *et al.*, 2004). More accurate protein quantification as well as characterisation of proteins in urine could be achieved with modern -omics technologies (Lin *et al.*, 2018).

Numerous studies have shown that NMR spectroscopy is a fundamental technology for metabolic profiling (Takis *et al.*, 2019; Vignoli *et al.*, 2019). Several techniques-experiments have been developed for protein identification/quantification in biofluids, mostly based upon diffusion coefficients (Lee *et al.*, 2020) and transverse relaxation times (T_2) (Rastrelli *et al.*, 2009) modulation by the presence of proteins. These approaches require the setup of specific experiments/pulse sequencies and there are not straightforward to automated analysis/interpretation.

The standardised pipeline of urine NMR-based metabolomics consists of a standard 1D ^{1}H NMR accompanied with a pseudo-2D *J*-Res experiment, where the latter is used for facilitating metabolites signals assignment (Dona *et al.*, 2014). This set of experiments allows the large urine cohorts analyses with minimal cost. In the 1D ^{1}H NMR urine profiles, protein signals appear as broad peaks which at the spectral edges (0–0.5 ppm and 6–10 ppm) are perceived as the baseline distortions (**Fig. S1**). We have recently shown that the integration of the metabolite-scarce regions of standardized metabolomics NMR spectra could provide the absolute total protein concentration (Vuckovic *et al.*, 2021). Before integration, regions of interest need to be de-metabolised (i.e. metabolites' signals should be filtered out).

In *NMRpQuant*, we incorporated the automated demetabolization and integration of three spectral regions (bottom panels of. **Fig. S1**), which include protons from the methyl (–CH_3, 0.2–0.7 ppm) and amide/aromatics (AA) (–NH, –Ar, 8–10 ppm) regions (Vuckovic *et al.*,

2021). The selection of these regions is based on their very low (0.2–0.7 ppm) or scarce (8–10 ppm) population by metabolite ^{1}H NMR signals. Therefore, integrals of metabolite residuals after filtering are negligible to the main protein integral.

<u>It should be noted that</u>: (**i**) urine NMR spectra should be of high quality (Sands *et al.*, 2019), particularly regarding their baseline and phase correction, because integration of NMR regions containing methyl groups (i.e. 0.2–0.5 and 0.2–0.7 ppm) are baseline and phase correction sensitive. (**ii**) Some protein amide protons (–NH) are in chemical exchange with water and their integrals may be affected by water signal suppression pulse sequences. So, it is highly recommended (and incorporated into our software) to ***combine*** the calculated total protein concentration from the afore-mentioned spectral regions.

4. *NMRpQuant* platform

4.1 The general purpose

The main purpose of the platform is the automated calculation of total protein absolute concentration (mg/mL) in urine from the standard 1D ^{1}H NMR urine spectra. As previously shown (Vuckovic *et al.*, 2021), most reliable results are obtained by integrating the spectral ranges of 8–10 and 0.2–0.7 ppm, corresponding to parts of aromatic/amide (–NH) and methyl (–CH_3) protein protons, respectively. Before integration, spectral regions are automatically demetabolised to achieve more accurate integration of protein signals. To remove (i.e. filter) metabolites signals from the spectral regions, three automated filtering methods are implemented: **i**) NCD (NOESY CPMG difference) filtering, **ii**) modified SMolESY filtering and **iii**) protein signals extraction from the 0.2–0.5 ppm spectral region only (see details each filtering method details below). When the spectrum is demetabolised (by any implemented method), the automated integration of one up to three spectral regions takes place and the integral(s) is(are) translated into absolute concentration (mg/mL). *NMRpQuant* incorporates the calibration factors for the three spectral regions (based upon Bruker IVDr ERETIC reference signal previously described in (Vuckovic *et al.*, 2021)) that converts mM proton concentration into mg/mL protein concentration. If mM concertation is not known (no ERETIC), it allows the use of any defined reference signal. Finally, quantitation results are exported in readily available reports, that could be used for any bioanalytical/clinical research.
→ It should be noted that any mentioned function of our software in the following sections, could be downloaded/found at https://github.com/pantakis/NMRpQuant.

4.2 Metabolites ^{1}H NMR signals removal (filtering) methods

4.2.1 NCD method (optional)

To run NCD method (Vuckovic *et al.*, 2021), software requires Carr−Purcell− Meiboom−Gill (CPMG) (Carr and Purcell, 1954) 1D spectrum. Consequently, for NCD method user should have acquired the CPMG spectra with the same parameters as the standard 1D ^{1}H NMR.
NCD method is incorporated in *NMRpQuant* via the:

```
function loadCPMG
```

By the time user uploads CPMG spectra [function loadCPMG]), each CPMG spectrum is aligned to zero ppm (i.e. via the TSP signal, see SOPs in section 1) by the:

```
function Align_data
```

and the intensity data of each CPMG spectrum (CPMG_Y_cal) is subtracted by the corresponding data of the standard 1D spectrum (Standard_Y1D):

```
NCDspectra = Standard_Y1D - CPMG_Y_cal
```

An example of NCD filtering is given in **Fig. S2**.

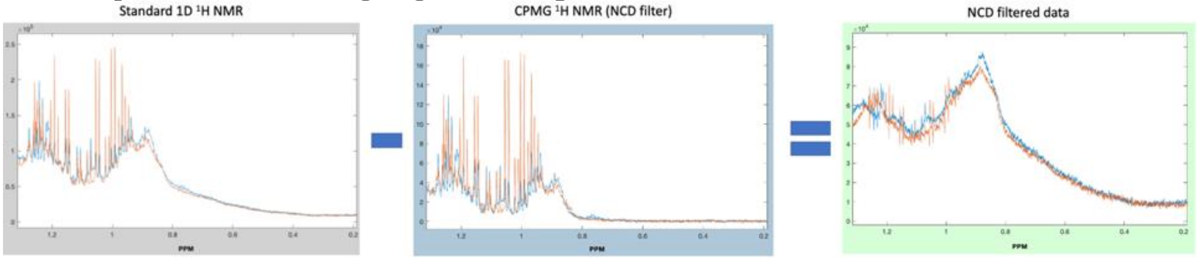

Figure S2. The NCD filtering process for two proteinuria ^{1}H NMR profiles. In the right panel the NCD filtered data are depicted. All spectra are focused on the protein methyl groups region, where in the NCD filtered data all small metabolites signals are depleted, so the integration of any aliphatic region provides the total protein in each urine sample.

4.2.2 SMolESY filtering

Small Molecule Enhancement SpectroscopY (SMolESY) (Takis *et al.*, 2020) was recently introduced for the computational suppression (https://github.com/pantakis/SMolESY_platform) of macromolecular signals from standard 1D spectra. A manually modified SMolESY method has been used for the small metabolites ^{1}H NMR signals suppression from the standard 1D urine NMR spectra (Vuckovic *et al.*, 2021). (**Fig. S3**).

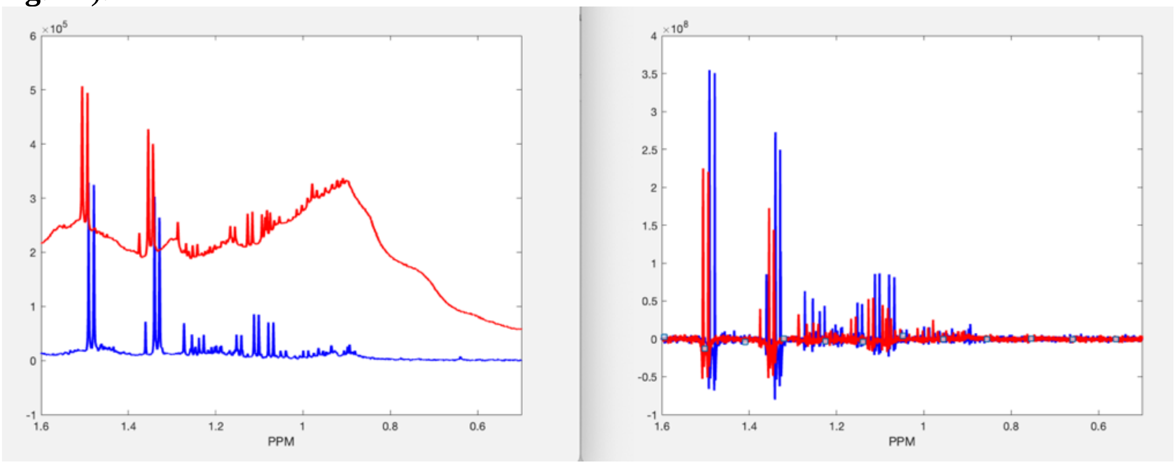

Figure S3. In the left panel there are two urine ^{1}H NMR spectra (from a patient with proteinuria (red) and one from a healthy subject) focusing on the aliphatic region. In the right panel are the SMolESY spectra where the protein background is depleted, and only the small metabolites profiles are further enhanced.

In this platform we implemented SMolESY filter, (function Process_lb_NMRdata) that automatically removes narrow metabolite lines from 1D urine spectrum (**Fig. S4**).

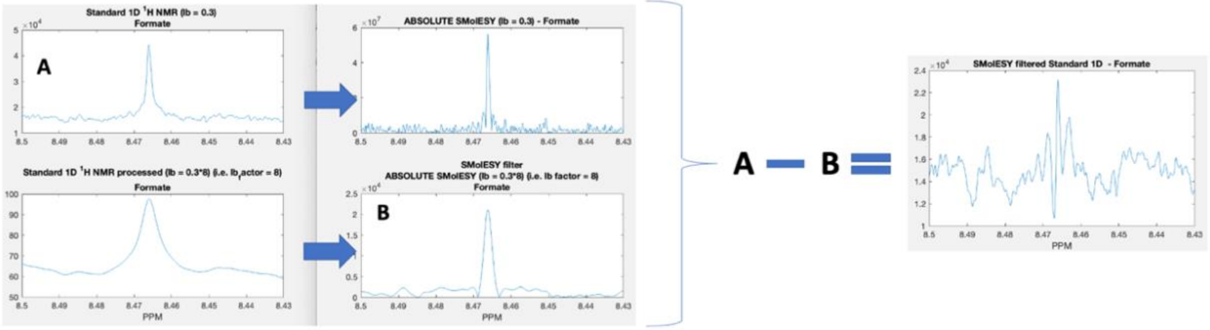

Figure S4. SMolESY filtered data production.

As shown in **Fig. S5**, the subtraction of the modified SMolESY data (i.e. SMolESY filter) from the standard 1D ^{1}H NMR spectra can deplete the sharp signals of small metabolites quite similar to the NCD.

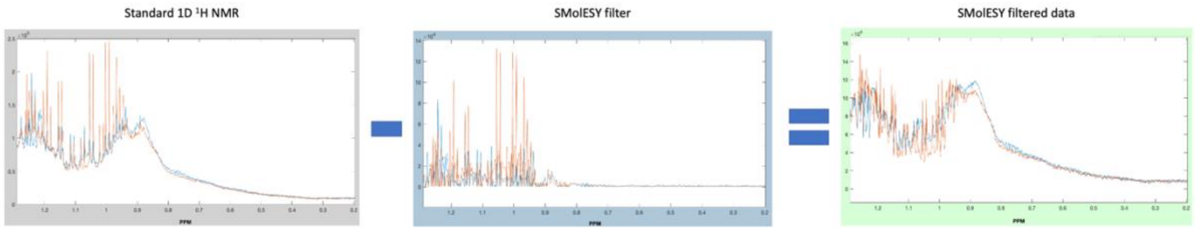

Figure S5. The SMolESY filtering process for two proteinuria ^{1}H NMR profiles. In the right panel the SMolESY filtered data are depicted. All spectra are focused on the protein methyl groups region, where in the SMolESY filtered data all small metabolites signals are depleted, so the integration of any aliphatic region provides the total protein in each urine sample.

SMolESY filters and the SMolESY based filtered ^{1}H NMR profiles are produced by the function:

```
function NMRpQuant_SMolESY
```

4.2.3 Protein (broad) signals extraction from 0.2–0.5 ppm spectral region

Protein (broad) signals extraction filter is a newly introduced filter, implemented in *NMRpQuant*. By selecting this filter, a linear interpolation fitting process takes place in the spectral region of 0.2–0.5 ppm, where the signals from a part of the aliphatic protein protons (–CH_3) resonate. Distinctive property of this region is infrequent appearance of very few narrow lines which tolerates simplified filtering.

The fitting process removes all spectral lines of small metabolites in that region (usually scarcely populated), and the area under the fitted line could be integrated, representing the total protein amount in the sample (**Fig. S6**).

The implemented function for protein signals fitting is:

```
function NMRpQuant_Baseline_filter
```

which encloses the function:

```
function base_fit_protein_linear
```

Initially, the main function applies a linear fitting employing the edge datapoints of the 0.2–0.5 ppm region (**top panel Fig. S6A**). After, the algorithm employs the negative residuals of the 1st fitting which may include sharp NMR signals of small metabolites. In the negative residuals (i.e. residuals multiplied by -1), the function finds all local maxima (i.e. humps which represent the edges of each sharp NMR signal) above spectral noise via MATLAB function findpeaks (https://uk.mathworks.com/help/signal/ref/findpeaks.html) (**middle panel Fig. S6A**). When all

maxima of the negative residuals are spotted, a new vector of x axis datapoints is constructed including maxima and edge points locations on x-axis. Then function base_fit_protein_linear linearly interpolates between each point via MATLAB function interp1 constructing the final baseline (**bottom panel Fig. S6A** and **Fig. S6B**).

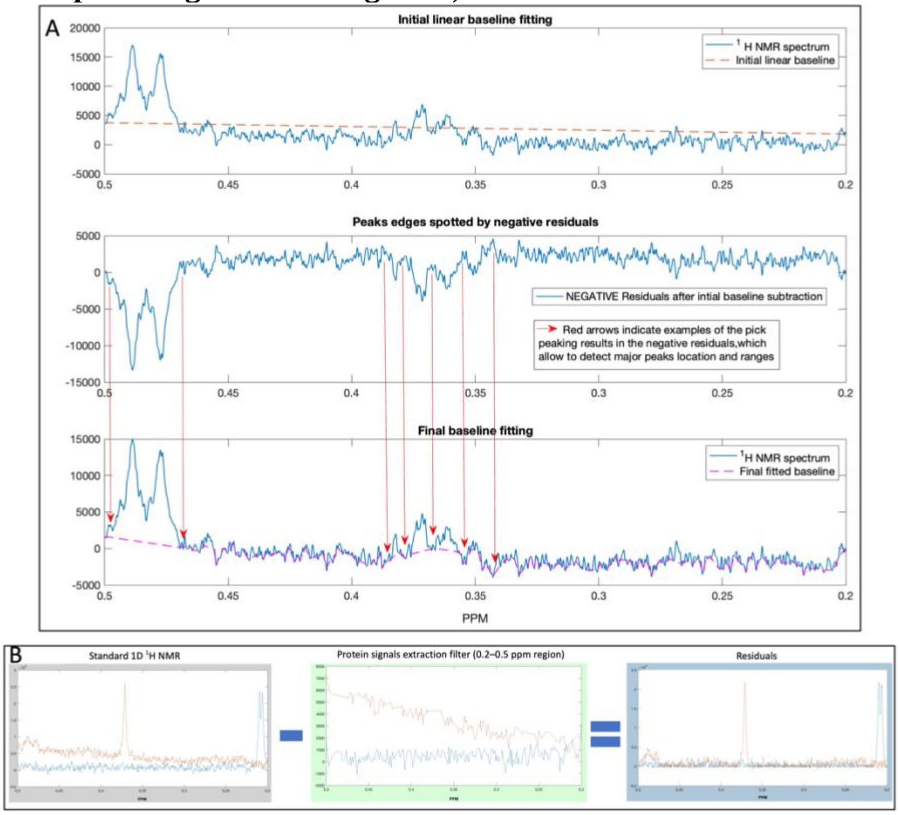

Figure S6. The protein signals extraction filter for 2 urine ^{1}H NMR profiles. (A) The algorithm's process for extracting protein signals via linear baseline fitting. (B) In the middle panel the filtered data are represented by the fitted protein signals (i.e. spectral baseline) and in the right panel the residuals of the fitting clearly show the removal of any sharp signal from small metabolites resonating in the region of 0.2–0.5 ppm.

4.3 Automated spectral regions integration – Total protein absolute quantification

As previously mentioned, *NMRpQuant* incorporates the automated integration of three spectral regions from metabolites' ^{1}H NMR signals filtered spectra, which consist of part of proteins methyl protons (i.e. 0.2–0.5 and 0.2–0.7 ppm) and part of the aromatic/amide protons (8–10 ppm). For NCD and SMolESY filtered NMR spectra, the algorithm could integrate up to three spectral regions function find_region_integrate, whereas for the protein signals extraction from the 0.2–0.5 ppm range software automatically integrates the corresponding region.

Table S1. Integral multiplies for mM to mg/mL conversion of total protein absolute quantitation (Vuckovic *et al.*, 2021).

Integrated spectral area (ppm)	0.2–0.5	0.2–0.7	8.0–10.0
K^{α} [(mg/mL/mM)]	2.7	1.05	0.53
ΔK^{b} [(mg/mL/mM)]	0.028	0.062	0.033

$^{\alpha}$K is a factor with each integral found in a given range (in units of mM) is multiplied to get total protein concentration in mg/mL.
$^{b}\Delta$K is the ± error factor with each integral found in a given range (in units of mM) is multiplied to get total protein concentration in mg/mL.

Following the above-mentioned steps, the produced integrations results represent total urine protein concentration in arbitrary units (a.u.), which could be exported without proceeding to

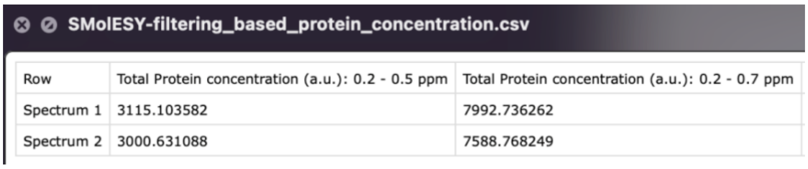

SMolESY-filtering_based_protein_concentration.csv

Row	Total Protein concentration (a.u.): 0.2 - 0.5 ppm	Total Protein concentration (a.u.): 0.2 - 0.7 ppm
Spectrum 1	3115.103582	7992.736262
Spectrum 2	3000.631088	7588.768249

Figure S7. Total protein concentration via integration without applying calibration curves (a.u.) output .csv file structure.

any absolute quantification (**Fig. S7**).

For the total protein absolute quantitation (i.e. mg/mL), the software—based upon a ERETIC signal—converts integrals into mM concentration and via specific calibration (multiplication) factors (**Table S1**), concentrations are translated them into mg/mL (**Fig. S8**) of total urinary protein (Vuckovic *et al.*, 2021) for the three integrated spectral regions, regardless of the applied filtering method (**Fig. S8A**). Moreover, *NMRpQuant* allows the

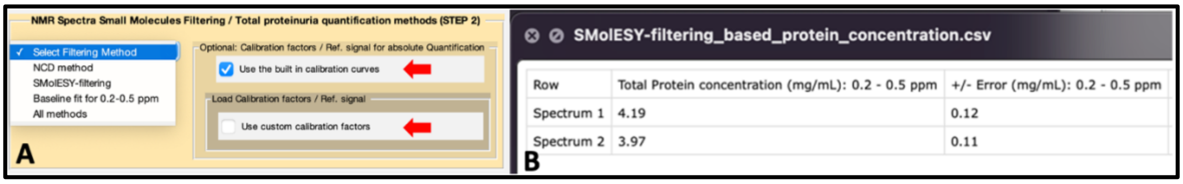

Row	Total Protein concentration (mg/mL): 0.2 - 0.5 ppm	+/- Error (mg/mL): 0.2 - 0.5 ppm
Spectrum 1	4.19	0.12
Spectrum 2	3.97	0.11

Figure S8. (A) By selecting one of the options indicated by red arrows total protein concentration could be translated into absolute values in mg/mL. (B) Absolute total protein concentration in mg/mL output .csv file structure.

implementation of any other reference signal and (if needed) custom calculated calibration factors for the above-mentioned regions for the absolute quantitation of total protein in urine samples. For more details, please see Section 5.3.

5. Guidelines plus detailed Graphical User Interface (GUI) description

NMRpQuant is integrated in a user-friendly graphical user interface (any mentioned function of our software in the following sections and a HOW-TO-USE DEMO video can be found at: https://github.com/pantakis/NMRpQuant), allowing the user to automatically calculate the total urine protein via the ^{1}H NMR urine profiles by following 5 steps, consisting of spectra interactive plots/visualization options and exporting results (**Fig. S9**). It should be noted that GUI incorporates plotting handles toolbar for zooming (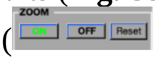

), pan and data-tips (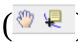) options.

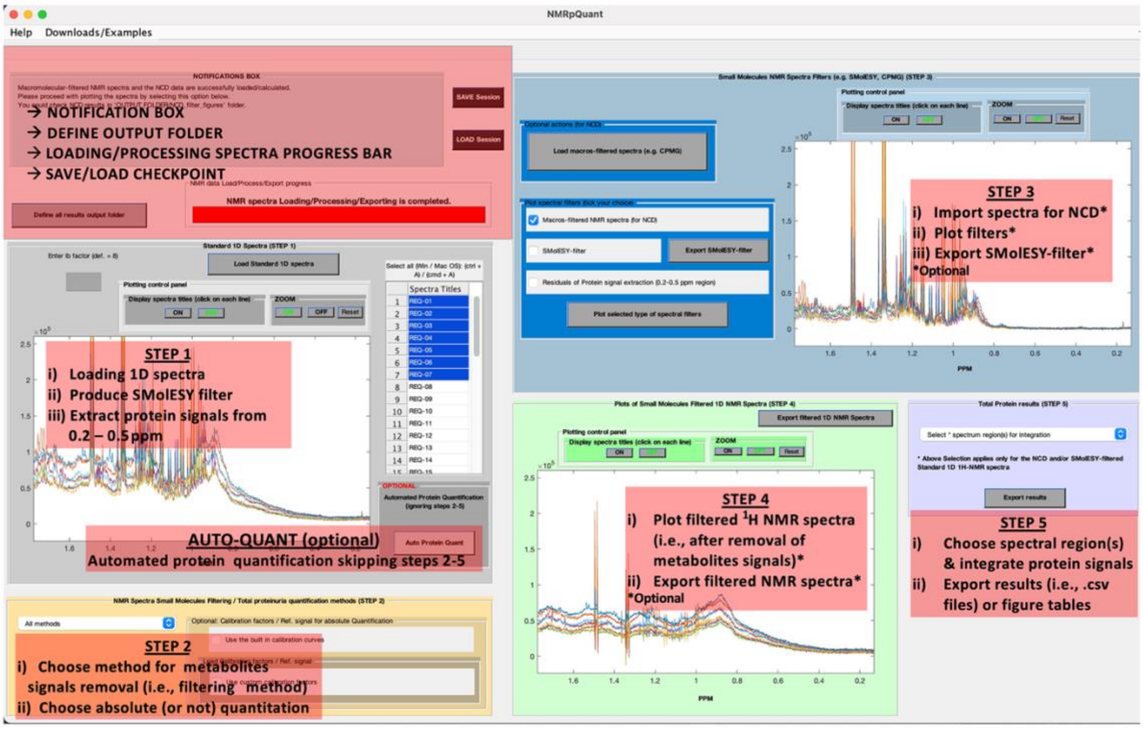

Figure S9. *NMRpQuant* GUI description/features. GUI consists of 5 steps highlighted by red font text boxes.

5.1 NOTIFICATIONS BOX – Output folder – Spectra loading/processing progress bar

The GUI consists of a NOTIFICATIONS BOX, where user is notified on real time about all running processes (and/or if there is a technical problem), such us the loading/processing of NMR spectra, preparation of metabolites signals removal filters, integration of spectral regions, exporting spectral data/integration results, as well as the directories that each kind of results is located (**Fig. S10**). In addition, the progress of loading/processing NMR spectra as well as automated filters production is indicated by a progress bar (**Fig. S10**). Finally, the user by pressing the button 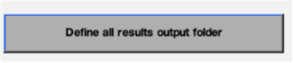

could define the parent output folder, where are data/results will be exported.

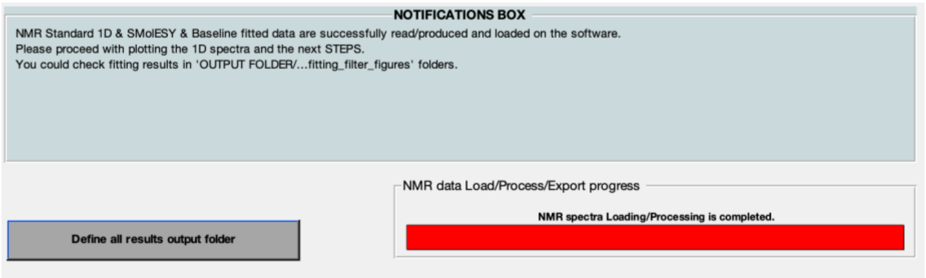

Figure S10. Notification box and general processes progress bar always inform user about the stage of software calculation/figures/results exportations as well as where each set of outcomes is deposited in the output folder.

5.2 STEP 1

In the STEP 1 of the algorithm (**Fig. S11**), spectra files are loaded/read by *NMRpQuant*, while two out of the three implemented filtering methods are automatically applied (i.e. SMolESY based filtering and protein signals extraction from 0.2 – 0.5 ppm spectral region), when loading the standard 1D ^{1}H NMR spectra. For the SMolESY-based filters production (see Section 4.2.2), user could adjust line broadening (lb) factor by entering a value here: 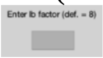

(function lb_factor). This factor will be used to multiply the applied line-broadening value (i.e. *lb* value) of the original processed 1D ^{1}H NMR spectra and re-process (i.e. perform Fourier-transform applying new *lb* value) their FIDs. From our initial and validation results (see Section 6), the 8 times higher initial *lb* value (i.e. **lb factor default value = 8**) is enough to provide highly correlated total protein results with clinical analyses (see Section 4.2.2 and Section 6.1). Re-processing of NMR spectra applying the new *lb* values is performed by the function Process_lb_NMRdata, by the time user presses the button: 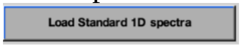

(function load1D). Consequently, *lb factor*—optionally—should be inserted before loading NMR spectra.

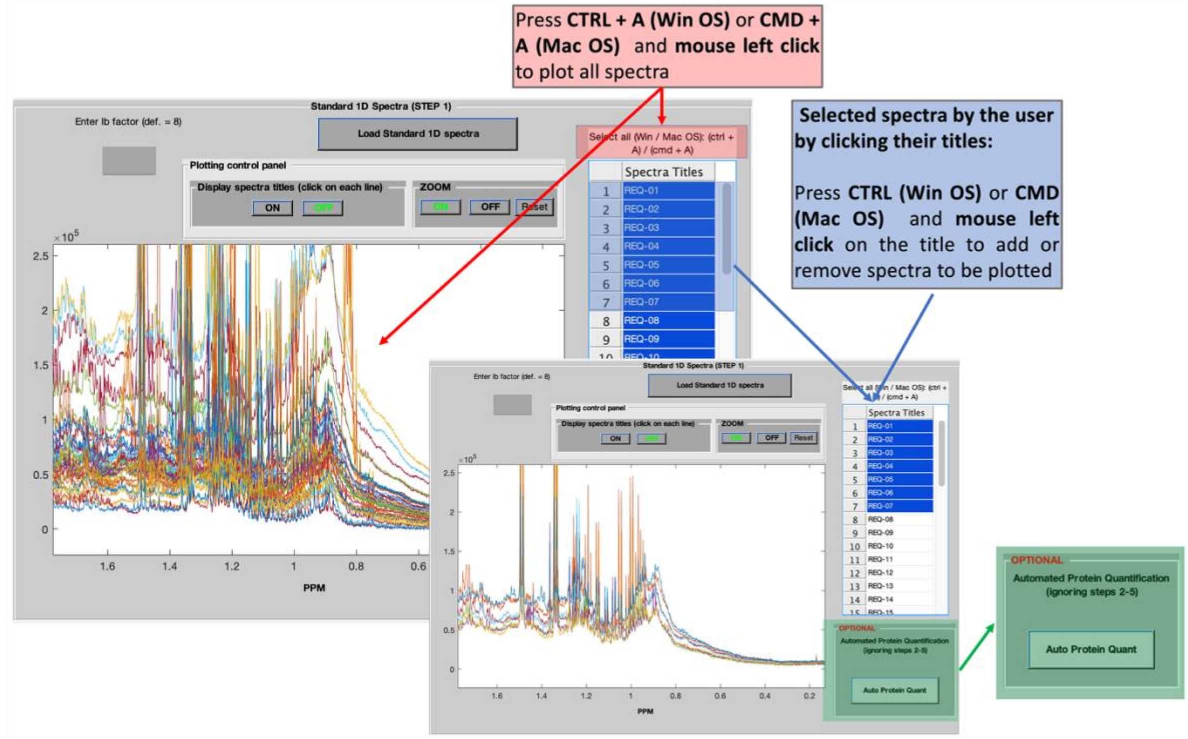

Figure S11. STEP 1 of the NMRpQuant GUI consists of: i) adjustment of SMolESY filters (optional) by inserting an *lb factor* value (default **validated** value = 8), ii) loading/plotting 1D ^{1}H NMR urine spectra and iii) (**optionally**) the automated total protein (absolute or not) quantification based upon SMolESY-filtering.

Additionally, when loaded, all spectra are automatically plotted, while activating (by pressing **ON** button) "display spectra titles" panel, user could "left mouse-click" on each spectral line to highlight and get the corresponding spectrum title (**Fig. S11**) (default is **OFF**). In this step, all titles of the NMR spectra are loaded in the GUI table (pointed by the blue arrows in **Fig. S11)** and the user could select by "left mouse-click" any spectrum by its title to be plotted, while for adding or removing selected spectra, the user could hold down CTRL (for Windows) or CMD (for macOS) + "left mouse-click" on any spectrum title. To select and re-plot all spectra user could press CTRL + A(for Windows) or CMD + A (for macOS). For further details about spectra folders' structure and supported NMR files are described in Section 5.7.

Finally, the user could select (by pressing the button: 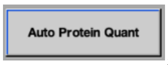

, see **Fig. S11)** the automated total urinary protein (absolute or not based upon the presence of ERETIC signal, see Section 4.3) quantification via SMolESY filtering method for metabolites signals removal (see Section 4.2.2).

5.3 STEP 2

In the STEP 2 of *NMRpQuant* (**Fig. S12A**), user could select one or all implemented methods for metabolites ^{1}H NMR signals removal (i.e. small molecules filtering method) from a drop-down list menu. As previously mentioned, the integrated filtering methods are: i) NCD method (where an extra type set of ^{1}H NMR spectra, e.g. CPMG, is mandatory to be uploaded to the software in STEP 4), ii) SMolESY based filtering and iii)| the extraction of protein signals from the 0.2–0.5 spectral region via fitting functions.

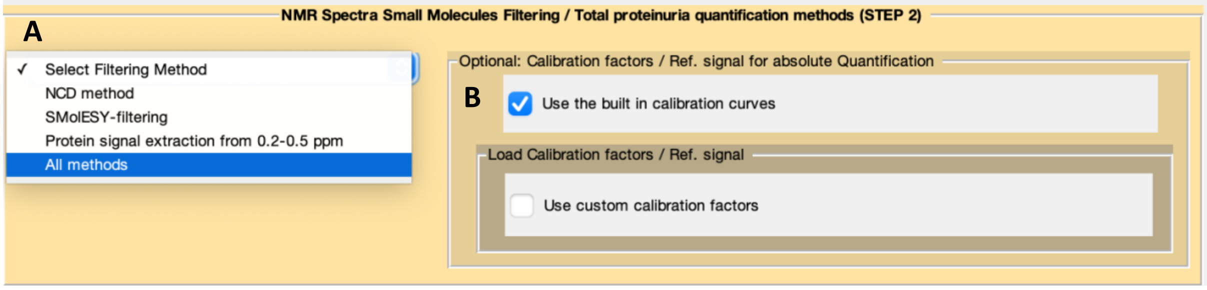

Figure S12. (A) Selection menu for choosing the filtering method for ^{1}H NMR signals from small urine metabolites removal. User could select NCD or SMolESY based filtering or protein signals extraction from the 0.2–0.5 ppm spectral region or all methods (function Method). NCD method is optional. **(B)** By selecting one of the options total protein concentration could be translated into absolute values (mg/mL).

Following the selection of any method for metabolites signals removal, user could translate integrals (see Section 4.3) into total urinary protein absolute concentration (i.e. mg/mL) by selecting this option as indicated in **Fig. S12B** (function UseBuiltInCurves).

By selecting build in calibration (i.e. default method/parameters for absolute quantitation), *NMRpQuant* converts each integral (a.u.) into mM concentration based upon ERETIC signal (see Section 4.3).

Afterwards, each concentration value per integrated region is translated into mg/mL of total urinary protein via previously calculated calibration (multiplication) factors [see (Vuckovic *et al.*, 2021)]. **Table S1 and Fig. S13** indicate all default calibration factors (Vuckovic *et al.*, 2021) for the three integrated spectral regions regardless of the applied filtering method, which are implemented in the “built in calibration curves” option (**Fig. S12B**).

TEMPLATE FOR LOADING NEW PARAMETERS FOR ABS QUANTIFICATION IN NMRpQUANT		
FACTORS	DETAILS	VALUES
Reference Signal Region for integration (ppm) (mandatory)	min	11.9
	max	12.1
Reference compound concentration (mandatory)	mM	10
Calibration factor for absolute quantification (leave empty if unknown: results will be in a.u.) (at least one value is mandatory)	0.2 - 0.5 ppm region	2.7
	0.2 - 0.7 ppm region	1.05
	8.0 - 10.0 ppm region	0.53
ERROR factors (leave empty if unknown: errors will not be printed)	0.2 - 0.5 ppm region	0.028
	0.2 - 0.7 ppm region	0.062
	8.0 - 10.0 ppm region	0.033

Figure S13. Excel file template for custom calibration factors / reference signals importation in the software. User should provide the spectral region (min – max values) to integrate for the reference signal (i.e. 11.9 – 12.1 for the ERETIC signal), the concentration that signal's integral represents and the calibration factor for at least one integration region of the software to translate reference signal's mM concentration to total protein concentration in mg/mL. All details of the factors values/calculation are included in ref: (Vuckovic *et al.*, 2021) and Table S1.

In case of the absence of the Bruker IVDr ERETIC signal, the user should load alternative reference signal region along with the default calibration factors (recommended) or any custom calibration factor [calculated as indicated by (Vuckovic *et al.*, 2021)] by selecting the 2nd option (i.e. "Use custom calibration factors", **Fig. S12B**) (function AskNEWCurve). When choosing to load new parameters for absolute quantification a dialog window pops up, to select an excel file with a specific structure (**Fig. S13**), whose template is provided with the software ("*TEMPLATE_Cal factors for absolute TOT PROTEIN quantification.xlsx*"). In the template, user should define:

i) the spectral region (min – max ppm values) of the reference compound signal to be integrated by the software
ii) the proton concentration in mM for the reference compound.
iii) our built-in calibration (plus error) factors (recommended) or custom calculated values by the user following the approach of (Vuckovic *et al.*, 2021) per region.

5.4 STEP 3

Fig. S14 depicts the STEP 3 panel of the *NMRpQuant* GUI. Initially, if NCD filtering method is selected, user should upload CPMG spectra by pressing the button Load macros-filtered spectra (e.g. CPMG), and the NCD filtered ^{1}H NMR spectra will be automatically produced (see Section 4.2.1).

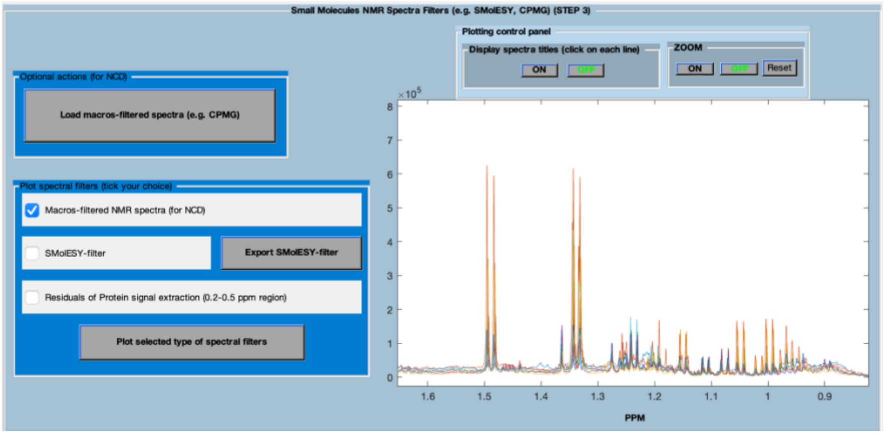

Figure S14. *NMRpQuant* GUI components for STEP 3, consist of loading NCD filter spectra (i.e. CPMG), selecting any produced/loaded filter to be plotted as well as the option to export each SMolESY-based filter per spectrum to a .csv file.

Otherwise, the user can select among SMolESY-filter and Residuals of Protein signal extraction from 0.2–0.5 ppm region and press Plot selected type of spectral filters (function PlotFilters) to plot the corresponding filters (**Fig. S15–S17**). In addition, modified SMolESY NMR data (i.e.

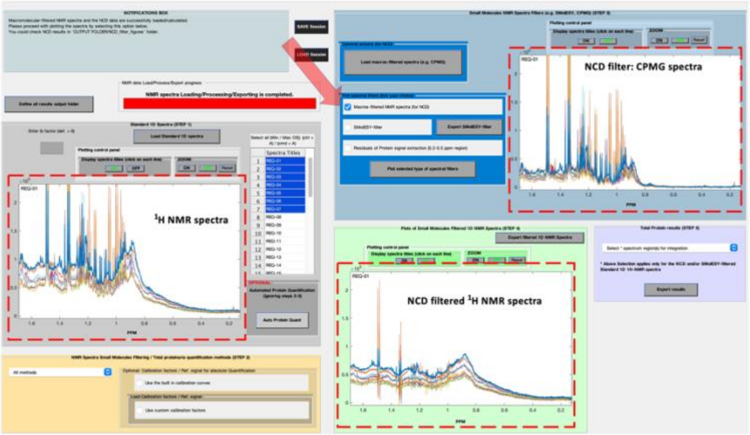

Figure S15. The case of selecting/plotting NCD filters (i.e. CPMG spectra) and the corresponding ^{1}H NMR NCD filtered data.

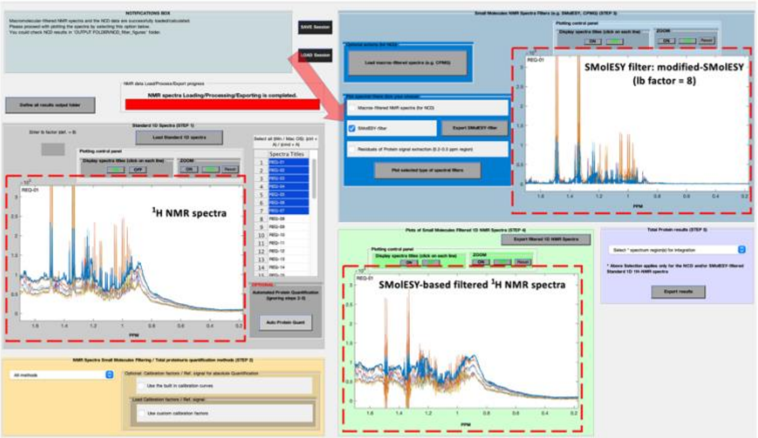

Figure S16. The case of selecting/plotting SMolESY filters (i.e. modified SMolESY using lb factor = 8) and the corresponding ^{1}H NMR SMolESY-based filtered data.

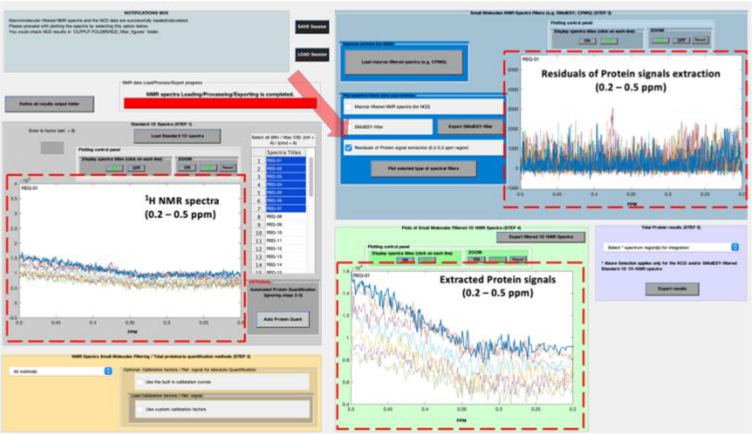

Figure S17. The case of selecting/plotting the residuals of the protein signals extraction from the 0.2–0.5 ppm spectral region and the extracted protein signals from the ¹H NMR data.

SMolESY-filter) could each one be exported to a .csv file per spectrum by the button: 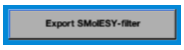

(function ExportSMolESY). Similar to STEP 1 (see Section 5.2), activation of "display spectra titles" panel, allows for highlighting/displaying each spectral line/spectrum title by "left mouse-clicking" on each line.

5.5 STEP 4

STEP 4 mainly consists of the plotting of the ¹H NMR spectra without the metabolites' 1H NMR signals (i.e. metabolites' filtered ¹H NMR spectra) (**Fig. S18**). The plot is automatically

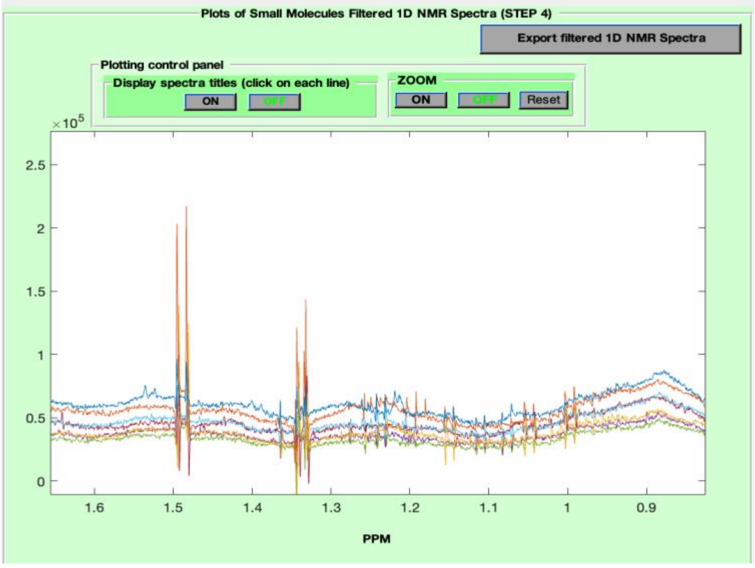

Figure S18. GUI's STEP 4 components consist of a plotting panel for the ¹H NMR spectra with suppressed signals from small metabolites (filtered spectra) as well as the option to export each filtered spectrum into a .csv file.

produced by the time user selects one filter to be plotted in STEP 3. In addition, user could export to .csv each filtered ¹H NMR spectrum from metabolites' signals by pressing the button: 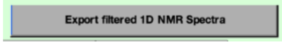

(function ExportFILTEREDgeneral).

5.6 STEP 5

The last step of the GUI is the automated integration of one up to three spectral regions that include parts of the total proteins' methyl and/or aromatic/amide protons (**Fig. S19**), previously

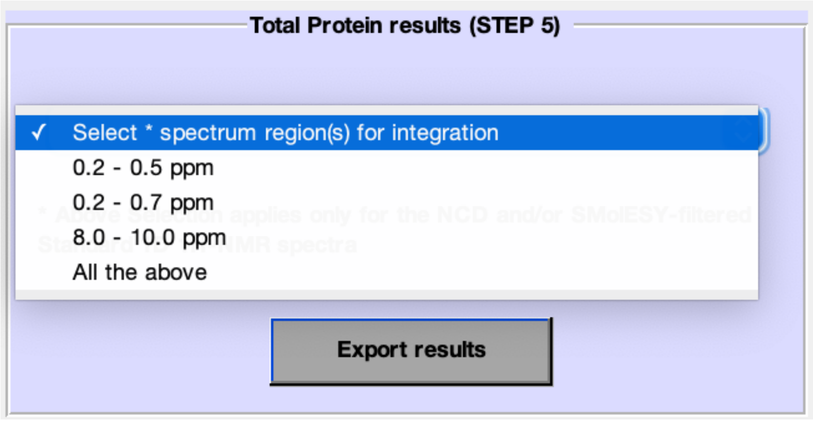

Figure S19. GUI's STEP 5 components consist of a drop-down list for choosing ^{1}H NMR spectral regions with suppressed signals from small metabolites (filtered spectra) to integrate, the calculation of integrals or absolute total urinary protein concentration and finally the exportation of the results into a .csv file.

described in Section 4.3. The main components of this step are a drop-down list for selecting the filtered from metabolites' signals spectral regions to integrate (**Fig. S20**) and calculate the integrals and/or the absolute concentration (function ChooseREGIONS_Int) of total urinary protein in mg/mL in each urine sample, provided that the user has selected this option in STEP 2. Finally, integration/quantification results are printed into tables—that appear as popped up figures that could be copied by the user— and exported to a .csv file by pressing the button 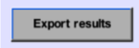

(function ExportRESULTS) .

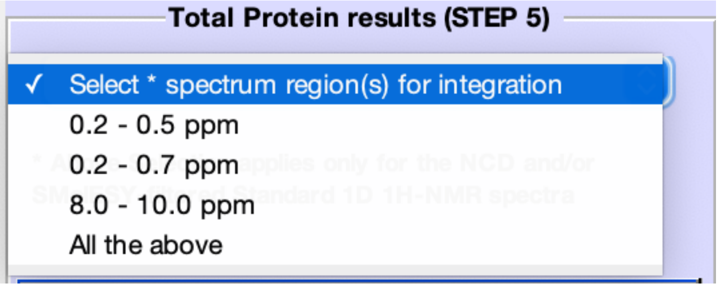

Figure S20. Selection menu of spectral regions for integration (function ChooseREGIONS_Int). This selection applies only when NCD or SMolESY filtering or all methods are selected. Namely, if protein signals extraction from 0.2–0.5 ppm filter is selected user can immediately press the 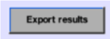

button. When selecting "All the above" for absolute quantification, the combination (i.e. average) of all calculated concentration values based on each spectra region is exported, too.

5.7 Prerequisites/Important notes for successful implementation of *NMRpQuant*

5.7.1 NMR spectra preparation/input files

→*NMRpQuant* is built to read Bruker NMR raw data.

→**All standard 1D ^{1}H-NMR urine spectra** (e.g. 1D-NOESY) should be in one parent folder as indicated in **Fig. S21** (Spectra Parent Folder). Please note that **non-spectral folders should NOT be inside the parent NMR data folder**.

→Under each spectrum (e.g. REQ_01, REQ_02 see **Fig. S21**) folder, there should by **ONLY** one experimental folder with a numerical name (e.g. 10).

→In each experimental folder, the software reads **ONLY** the "pdata/**1**" folder. The highlighted blue files (i.e 1r, 1i, fid etc.) in **Fig. S21** are the input files and should exist for each acquired spectrum in their corresponding folders as indicted in **Fig. S21** and are usually structured when a spectrum acquired via Bruker NMR instrumentation/acquisition software.

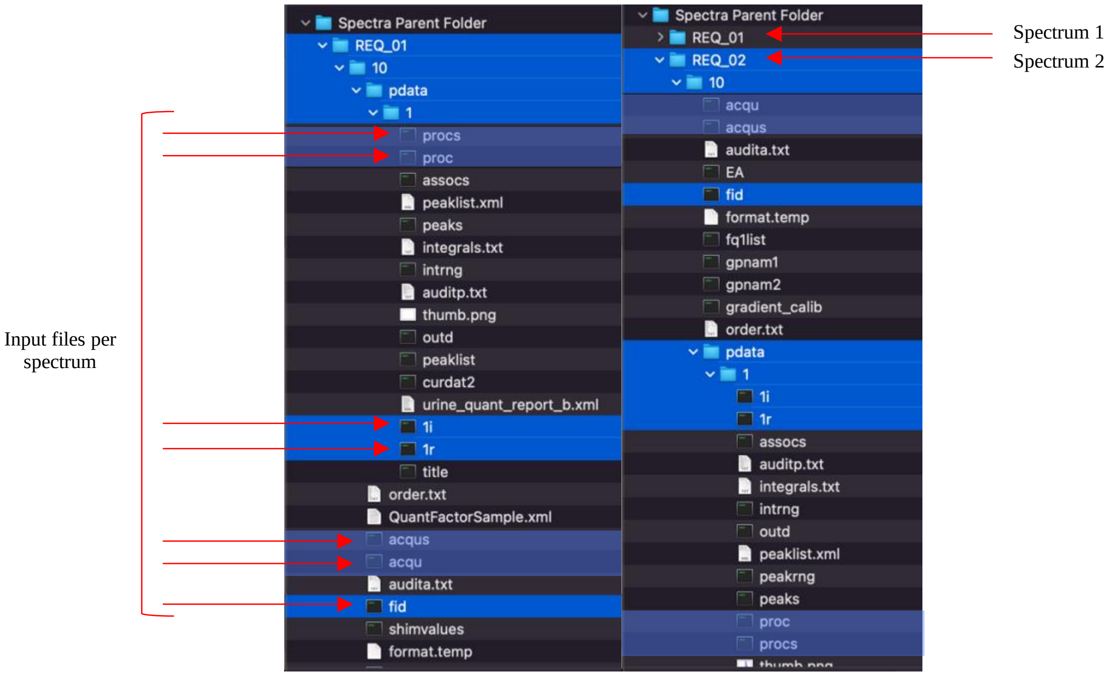

Figure S21. The structure of NMR raw data inside the parent folder. Blue highlighted are the mandatory files that are read by the software.

→The same requirements are needed when loading **NCD filter spectra** (e.g. CPMG spectra) (see Section 4.2.1 and Section 5.4), where CPMG spectra should be on a separate parent folder and should be acquired/processed with the same parameters as the standard 1D ^{1}H NMR, e.g. the same spectral width (sw), number of scans (ns), resolution (SI) etc. Each CPMG spectrum should have **the same title folder** (e.g. REQ_01, REQ_02 see **Fig. S21**) as its corresponding standard 1D ^{1}H NMR.

→Urine ^{1}H NMR spectra should be of high resolution (usually 64k to 128k datapoints) and quality, particularly regarding their baseline and phase correction, since integration of NMR regions containing methyl groups (i.e. 0.2–0.5 and 0.2–0.7 ppm) are baseline and phase correction sensitive (Sands *et al.*, 2019).

5.7.2 Urine samples preparation

→The urine samples should be treated/prepared according to the universally adopted standard operating procedures (SOPs) which are described in detail at Section 1.

5.7.3 Notes for SMolESY-based filtering – Protein extraction signals from 0.2–0.5 ppm region

→Both SMolESY filters and the extraction of protein signals from the 0.2–0.5 ppm spectral regions, are automatically produced when loading ^{1}H NMR spectra in STEP 1 of the GUI.

5.7.4 Notes for total urinary protein absolute quantification

→ Built in calibration factors for absolute quantification are based upon the ERETIC signal at 12.0 ppm that is produced in the urine NMR metabolomics platform by Bruker Biospin IVDr, whose integral corresponds to 10mM for one proton.

5.8 Save/Load an *NMRpQuant* session (save/load checkpoint)

NMRpQuant GUI incorporates 2 extra functionalities, where the user could save any running session to a ".mat" file (i.e. by pressing the button: 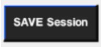

) and then reload it (i.e. by pressing the button: 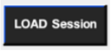

), so as to continue the total urinary protein quantification and/or explore ^{1}H NMR urine spectra.

6. *NMRpQuant*: performance in automated mode

The number of urine samples and the different cohorts that were used for *NMRpQuant* calibration/validation are described in the following sections as well as in the **Scheme S1**.

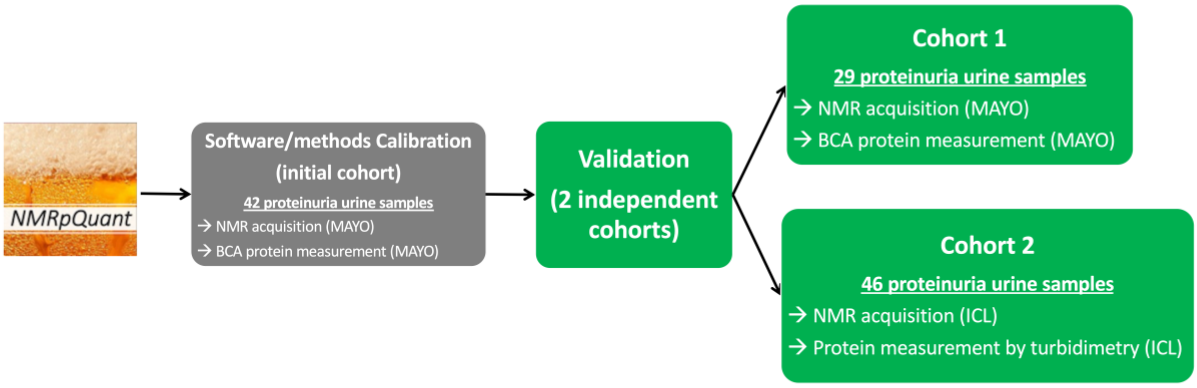

Scheme S1. Number of proteinuria samples/cohorts used for *NMRpQuant* calibration/validation.

6.1 Initial urine samples cohort: NMR vs BCA total protein quantification results

Automated integration of protein signals via all available filtering methods in *NMRpQuant*, was initially tested and fine-tuned based on the urine NMR spectra from 42 proteinuria patients (Vuckovic *et al.*, 2021) (**Scheme S1**). Integrals show a very high reproducibility for each integrated region (**Fig. S22**) among each filtering method ($R^2 > 0.99$).

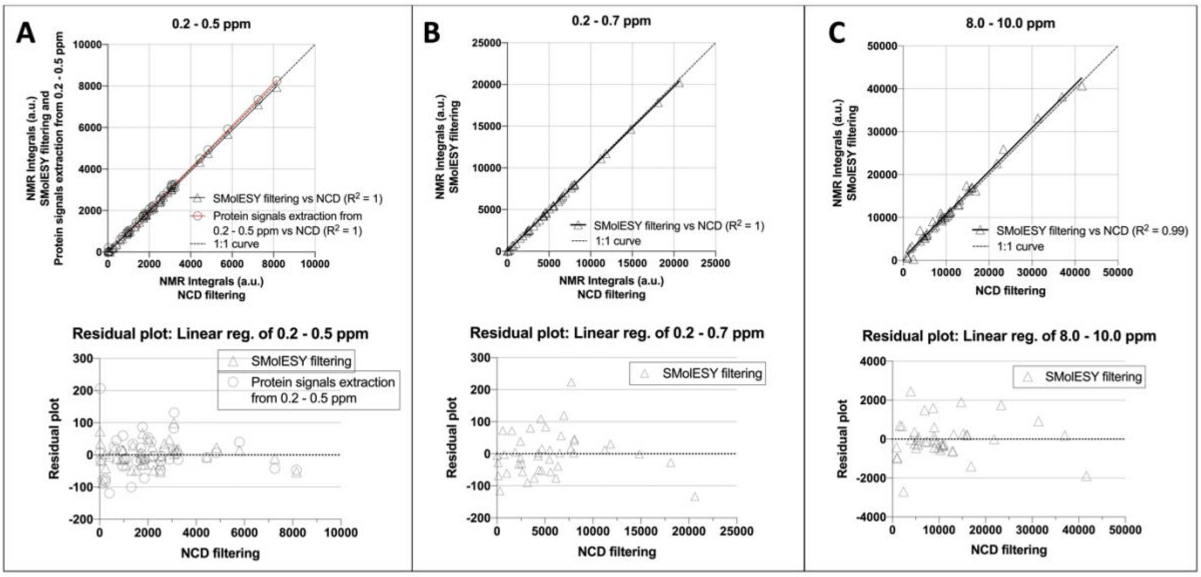

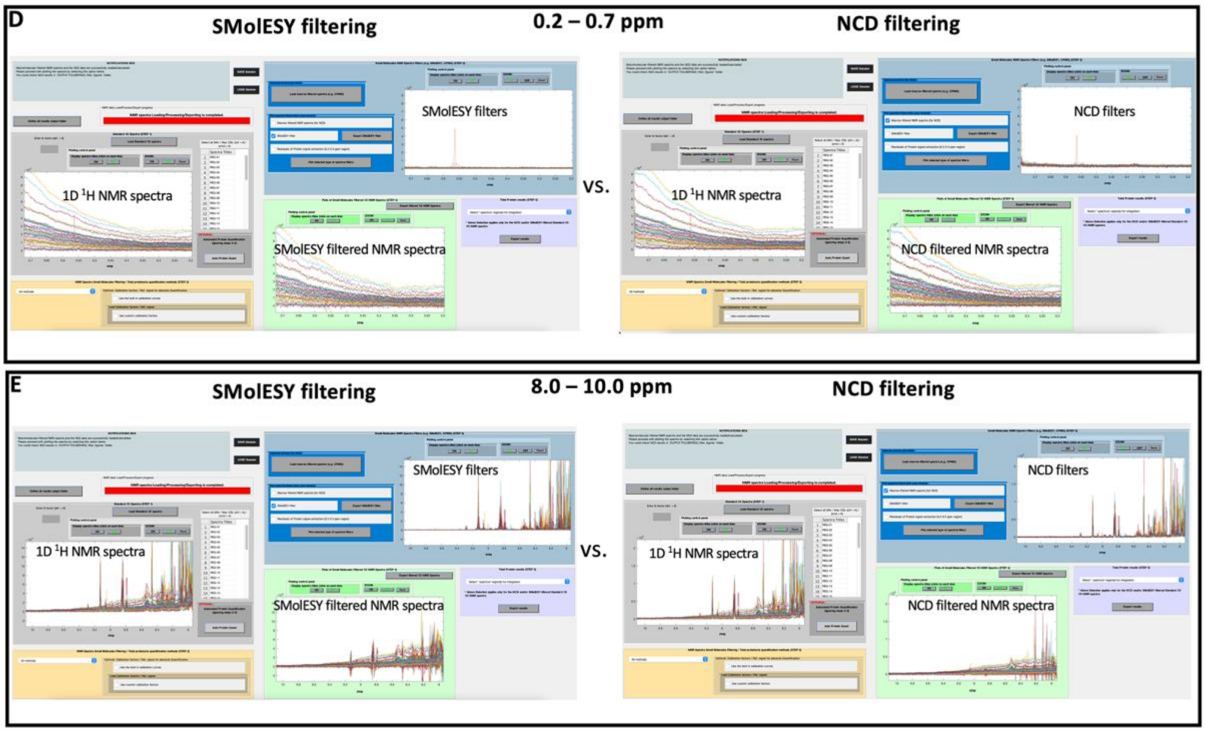

Figure S22. Comparison of the integration results among the integrated small metabolite signals filtering methods in *NMRpQuant*. (**A-C**) For the spectral regions 0.2–0.5, 0.2–0.7 and 8.0 – 10.0 ppm SMolESY vs NCD filtering methods provide almost identical results, however, SMolESY does not require extra NMR spectra acquisition. It should be noted that SMolESY filters are automatically produced by the default *lb_factor* = 8. (**D-E**) SMolESY and NCD filters/filtered data from the 42 urine spectra plots focusing on the aliphatic and -NH protein regions, respectively, (i.e. as being plotted in *NMRpQuant*).

Moreover, automated absolute quantification of total urine protein concentration (i.e. based upon the default calibration factors, see Fig. S13 in Section 5.3 and Table S1 (Vuckovic *et al.*, 2021)) are linearly correlated ($R^2 > 0.9$) to the concentrations from BCA assays, regardless of the integrated region/filtering method (**Fig. S23A-C**) and statistically coincide to the 1:1 curve.

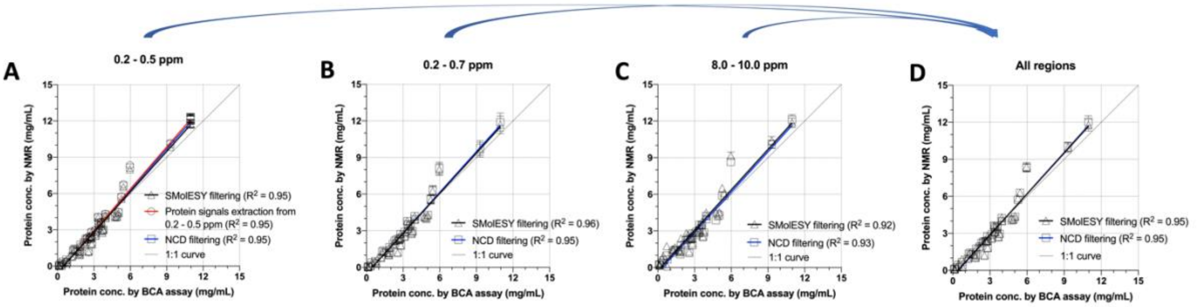

Figure S23. Comparison of the automated total protein absolute quantification based upon each integrated spectral region after applying all small metabolite signals filtering methods (A-C) and their combination (D) by *NMRpQuant* versus BCA total protein measured concentration (mg/mL). Urine samples and their spectra were previously used for the "manual" total protein quantification in (Vuckovic *et al.*, 2021).

Above results further validate the newly implemented automated approach for the SMolESY filter calculation, which previously (Vuckovic *et al.*, 2021) was accurately described by more complex equations minimization per spectrum. However, in *NMRpQuant* is highly simplified, via being tuned by the *lb factor* multiplier of the original *lb* values and the re-processing of the FID (i.e. via Fourier transformation) with the new *lb* values. It should be noted that, the above and following presented SMolESY filtered data are produced by the default value (i.e. *lb factor* = 8). In addition, **Fig. S23D** clearly confirms that the average of

the total protein concentration for each region (i.e. combination of multiple regions) per filtering method provides equally well results as each integral.

6.2 Multicentered validation urine samples cohorts: NMR vs (BCA & clinical methods) total protein quantification results

^{1}H NMR spectra of two independently collected urine samples cohorts (validation **cohort 1** and validation **cohort 2** consisted of 29 and 46 samples, respectively (**Scheme S1** in Section 6)) were acquired at different NMR centers and the protein content of the urine samples was determined by BCA and turbidimetry, respectively (details for the NMR samples preparation, spectra acquisition could be found in Section 1). The **cohort 1** was collected from patients suffering from focal segmental glomerulosclerosis (FSGS), whereas **cohort 2** was taken from patients suffering from viral infections, where proteinuria was recently associated with the severity of the infection (Huart *et al.*, 2021; Mohamed and Velez, 2021). For both cohorts, standard 1D ^{1}H NMR spectra were acquired,

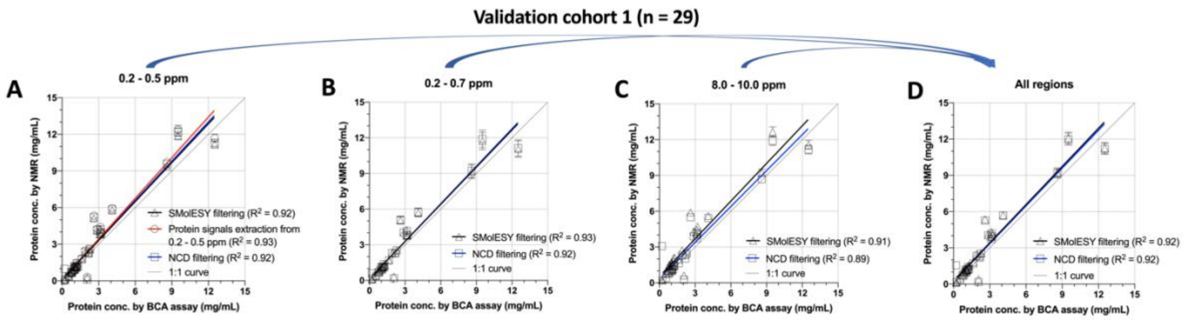

Figure S24. Comparison of the automated total protein absolute quantification by *NMRpQuant*, based upon (A-C) each integrated spectral region after applying all small metabolite signals filtering methods and (D) their combination versus BCA measured total protein concentration (mg/mL). Urine samples (n = 29) and their spectra are named as the validation **cohort 1**.

whereas CPMG spectra (i.e. NCD filter) were recorded only for validation **cohort 1**. Consequently, NMR based total urine protein absolute quantification was performed by applying all small metabolites signals filtering methods for the **cohort 1** (**Fig. S24**) and only SMolESY-based filters plus protein signals extraction from the 0.2–0.5 ppm were calculated

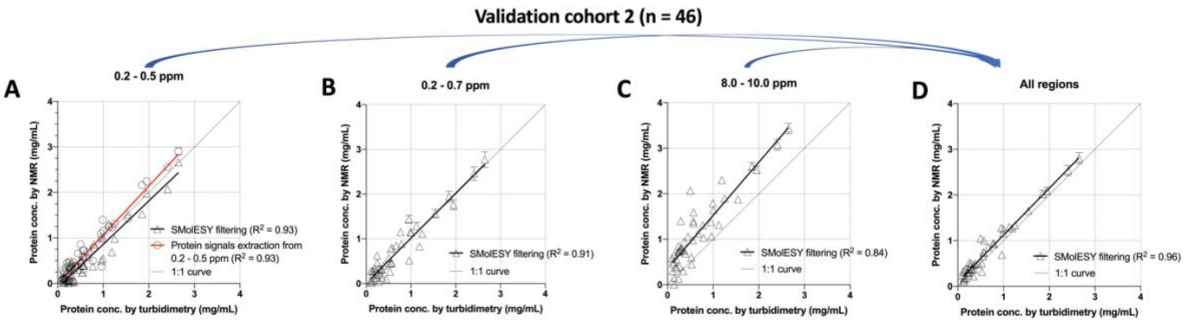

Figure S25. Comparison of the automated total protein absolute quantification by *NMRpQuant*, based upon (A-C) each integrated spectral region after applying only protein signals extraction from the 0.2–0.5 ppm region and SMolESY filtering methods and (D) their combination versus turbidimetrically measured total protein concentration (mg/mL). Urine samples (n = 46) and their spectra are named as the validation **cohort 2**.

for **cohort 2** (**Fig. S25**). Linear regression of the total protein quantitation for the urine samples of **cohort 1** (n = 29) by *NMRpQuant* (default *lb factor* value = 8 was applied for SMolESY

filtering) versus BCA results, showed a very good agreement, independently of the filtering method and the selected spectral region. It should be mentioned that few outliers, where NMR result deviates from BCA derived protein concentration, have been detected. The common feature of these outlier samples is high glucose level (glucose concentration in healthy urine should be very low), which may interact with protein quantification using BCA assay (Brown *et al.*, 1989). As expected, the combination of all NMR spectral regions quantification results (for both NCD and SMolESY filtering) provided a linear correlation with BCA results (**Fig. S24D**) with $R^2 = 0.92$. Regardless of very few outliers, *NMRpQuant* was in very good agreement with BCA, validating its overall performance on an independent cohort.

For the **cohort 2** (n = 46), an independent clinical method was followed to measure total urinary protein (i.e. turbidimetric approach) as well as urine NMR spectra were collected at a different site compared to **cohort 1** and the initial dataset described in Section 6.1. Based upon only SMolESY-based filtering method due to the lack of NCD filters (i.e. CPMG spectra were not acquired), *NMRpQuant* results showed a very good linear correlation with clinical measurements especially for the proteins' methyl protons ($R^2 \sim 0.93$) (**Fig. S25A-B**), whereas, quantification results based on the aromatic/amide protons integration (**Fig. S25C**) showed a lower correlation with clinical data and an overestimation of the protein concentration. This could be due to the amide protons sensitivity to chemical exchange and the presaturation pulse sequence used in NMR metabolomics pipeline which could be modulated by the pH of each sample. Nevertheless, as previously shown, (Vuckovic *et al.*, 2021) the combination of all spectral regions (including both parts of protein methyl and aromatic/amide protons) provided an excellent agreement and linear correlation ($R^2 = 0.96$) with turbidimetric results (**Fig. S25D**).

Table S2. ANOVA test results of linear regression curves coincidence. The comparison of the two validation cohorts' curves based upon the NMRpQuant total protein measurements (i.e. based upon the combination of both protein methyl and aromatic/amide regions integrals) versus clinical data indicates both slopes and intercepts of linear regression curves are not statistically different.

	**SMolESY filtering (Cohort 1: 29 samples)**	**SMolESY filtering (Cohort 2: 46 samples)**	**ANOVA (F-test) results**
**Best-fit values**			For the slopes
Slope	1.053	1.024	F = 0.04043
Y-intercept	0.2691	0.1004	DFn = 1
X-intercept	-0.2556	-0.09806	DFd = 71
1/slope	0.9498	0.9763	$P_{slope} = 0.8412$
**95% Confidence Intervals**			→The pooled slope equals: 1.051
Slope	0.9329 to 1.173	0.9551 to 1.093	→95% Confidence Intervals:
Y-intercept	-0.1856 to 0.7238	0.03941 to 0.1615	0.998 to 1.134
X-intercept	-0.7483 to 0.1641	-0.1663 to -0.03665	
**Goodness of Fit**			For the intercepts
R squared (R^2)	0.9232	0.9549	F = 1.665
Sy.x	0.8922	0.1403	DFn = 1
Equation	Y = 1.053*X + 0.2691	Y = 1.024*X + 0.1004	DFd = 72<br>$P_{intercept} = 0.2010$<br>→The pooled intercept equals: 0.1570<br>→95% Confidence Intervals: -0.02605 to 0.2843

Statistical analyses (ANOVA tests) for testing the coincidence of slopes and intercepts from the linear regression curves for each validation cohort (**Fig. 24D** and **Fig. 25D**), clearly indicate that both slopes and intercepts are not statistically different (**Table S2**, **Fig. 26**) (ANOVA analysis was performed by Prism 9, https://www.graphpad.com/) and the pooled curve statistically passes through the origin (0,0). These results further validate the wide applicability of *NMRpQuant* for protein quantitation, being in very good agreement with routine urine

analyses data. Overall, validation results based upon independently recorded NMR spectral data and independently clinically-measured total urinary protein results, indicate that the automated total urinary protein absolute quantification by *NMRpQuant* is in line with different routine approaches.

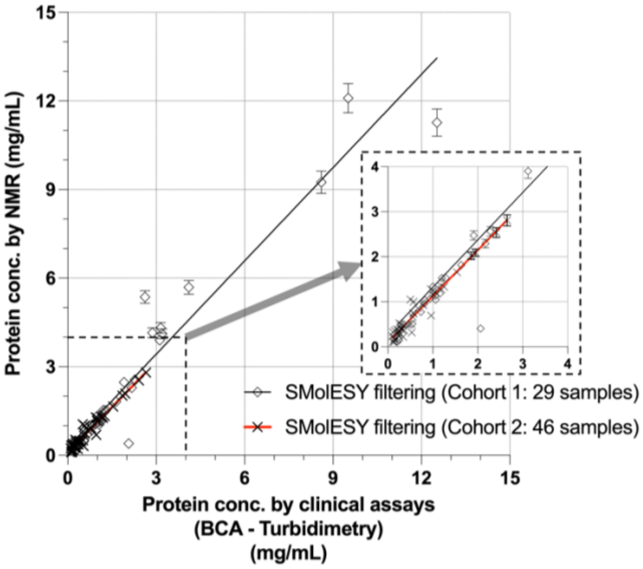

Figure S26. Linear regression curves of *NMRpQuant* versus clinically measured total urinary protein in the two validation cohorts' urine samples. ANOVA tests confirm both curves' slopes/intercepts statistical coincidence (**Table S2**). The two linear curves are based upon the NMRpQuant total protein measurements (i.e. based upon the combination of both methyl and aromatic/amide regions integrals) versus clinical data.

7. Availability of Raw NMR data and biochemical protein measurements – Input/Output files

→ All spectral raw NMR data from the initial cohort (n = 42) that was used to test/optimize NMRpQuant (see Section 6.1) and previously described in (Vuckovic *et al.*, 2021) as well as the total protein measured concentration for each urine sample *via* BCA assay could be found at https://doi.org/10.6084/m9.figshare.18737189.v1 repository. Both standard 1D (i.e. noesy1d) and CPMG (i.e. NCD filters) ^{1}H NMR profiles are provided to any user for testing *NMRpQuant*.

→ The total protein concentration values (both spectral integrals in a.u and mg/mL), all outputs (both figures and spectral data) regarding SMolESY-based and protein signals extraction filters for the removal of metabolites signals and their corresponding filtered data along with NCD filtered data, applied on the 42 urine samples ^{1}H NMR spectra, could be found at https://doi.org/10.6084/m9.figshare.18737372.v1 repository. All output data/files are provided to any user for testing/validating *NMRpQuant* output results for the 42 urine samples initial cohort (see Section 6.1).

8. Technical Requirements

8.1 MATLAB dependencies – Operating Systems

→*NMRpQuant* was built in MATLAB 2021b (MathWorks®) and it is fully functional in MATLAB 2021 and above.
→*NMRpQuant* is licensed under the GNU General Public License v3.0
→The software requires 'Signal Processing Toolbox' if run via MATLAB computing platform.
→To avoid MATLAB dependencies, *NMRpQuant* is also compiled for both Windows (.exe) 10 (and above) and macOS (.app) Sierra (and above) operating systems (OS) and could be directly installed in any of the two OS, without requiring any MATLAB license.

8.2 Recommended computational resources

→*NMRpQuant* requires >4gb RAM, an Intel or Apple Silicon processor.
→It is recommended to load up to 200 urine samples spectra per run to avoid any RAM overloading.
→For smooth experience of *NMRpQuant* GUI application, the minimum display requirements are 13-inch display with minimum resolution of full HD (1920x1080).

9. Supplementary References

Bouatra,S. *et al.* (2013) The human urine metabolome. *PLoS One*, **8**, e73076.

Brown,R.E. *et al.* (1989) Protein measurement using bicinchoninic acid: elimination of interfering substances. *Anal. Biochem.*, **180**, 136–139.

Carr,H.Y. and Purcell,E.M. (1954) Effects of diffusion on free precession in nuclear magnetic resonance experiments. *Phys. Rev.*, **94**, 630–638.

Dona,A.C. *et al.* (2014) Precision High-Throughput Proton NMR Spectroscopy of Human Urine, Serum, and Plasma for Large-Scale Metabolic Phenotyping. *Anal. Chem.*, **86**, 9887–9894.

Huart,J. *et al.* (2021) Proteinuria in COVID-19: prevalence, characterization and prognostic role. *J. Nephrol.*, **34**, 355–364.

Lamb,E.J. *et al.* (2009) How should proteinuria be detected and measured? *Ann. Clin. Biochem.*, **46**, 205–217.

Lee,J. *et al.* (2020) High-Resolution Diffusion Measurements of Proteins by NMR under Near-Physiological Conditions. *Anal. Chem.*, **92**, 5073–5081.

Lin,L. *et al.* (2018) Fast quantitative urinary proteomic profiling workflow for biomarker discovery in kidney cancer. *Clin. Proteomics*, **15**, 42.

Mohamed,M.M.B. and Velez,J.C.Q. (2021) Proteinuria in COVID-19. *Clin. Kidney J.*, **14**, i40–i47.

Rastrelli,F. *et al.* (2009) Seeing through macromolecules: T2-filtered NMR for the purity assay of functionalized nanosystems and the screening of biofluids. *J. Am. Chem. Soc.*, **131**, 14222–14224.

Sands,C.J. *et al.* (2019) The nPYc-Toolbox, a Python module for the pre-processing, quality-control and analysis of metabolic profiling datasets. *Bioinformatics*, **35**, 5359–5360.

Takis,P.G. *et al.* (2017) Deconvoluting interrelationships between concentrations and chemical shifts in urine provides a powerful analysis tool. *Nat. Commun.*, **8**, 1662.

Takis,P.G. *et al.* (2020) SMolESY: an efficient and quantitative alternative to on-instrument macromolecular 1H-NMR signal suppression. *Chem. Sci.*, **11**, 6000–6011.

Takis,P.G. *et al.* (2019) Uniqueness of the NMR approach to metabolomics. *TrAC Trends Anal. Chem.*, **120**, 115300.

Vignoli,A. *et al.* (2019) High-Throughput Metabolomics by 1D NMR. *Angew. Chemie - Int. Ed.*, **58**, 968–994.

Vuckovic,I. *et al.* (2021) 1H Nuclear Magnetic Resonance Spectroscopy-Based Methods for the Quantification of Proteins in Urine. *Anal. Chem.*, **93**, 13177–13186.

Yılmaz,F.M. *et al.* (2004) Automated Turbidimetric Benzalkonium Chloride Method for Measurement of Protein in Urine and Cerebrospinal Fluid. *Clin. Chem.*, **50**, 1450–1452.
